# Clinical characteristics of foreign-imported COVID-19 cases in Shanghai, China

**DOI:** 10.1080/22221751.2020.1766383

**Published:** 2020-06-09

**Authors:** Xu-hui Liu, Shui-hua Lu, Jun Chen, Lu Xia, Zong-guo Yang, Stratton Charles, Yang Yang, Yun Lin, Hong-zhou Lu

**Affiliations:** a Shanghai Public Health Clinical Center, Fudan University, Shanghai, People’s Republic of China; b Shanghai Zhongshan Hospital, Fudan University, Shanghai, People’s Republic of China; c TB Center, Shanghai Emerging and Re-emerging Institute, Shanghai, People’s Republic of China; d Vanderbilt University School of Medicine, Nashville, Tennessee

**Keywords:** COVID-19, SARS-CoV-2, imported case, clinical character, prevalence

## To the Editor,

In December 2019, a nationwide coronavirus 2019 (COVID-19) epidemic began in China and was finally controlled in early March 2020 [1, 2]. Few new domestic cases have been reported since late March 2020. Administrative screening for COVID-19 was initiated for people entering China beginning in March 2020. As travel to China from foreign countries has increased, imported cases of COVID-19 have also sharply increased [1]. Thus, China is facing new challenges in the fight against COVID-19.

A cross-sectional study of 58 patients aged 16–75 years and confirmed to have COVID-19 was conducted at Shanghai Public Health Clinical Center (SHPHC). COVID-19 was diagnosed according to World Health Organization interim guidelines. According to hospital records, the patients were admitted between 5 March and 22 March 2020. The ethics committee of SHPHC reviewed and approved the study procedure.

The clinical characteristics of patients with imported COVID-19 were reviewed using descriptive clinical epidemiological methods including clinical symptoms, exposure history, chest computed tomography (CT) imaging findings, and type of oxygen support required Anti-COVID-19 antibody, hematologic test, clinical chemistry test, coagulation test, and immune and inflammatory indices were obtained from the medical record system and the sampling time was no more than 24 h after admission.

Severe cases were defined as having at least one of the following criteria: (1) breath rate ≥30/min; (2) pulse oximeter oxygen saturation (SpO_2_) ≤93% at rest; (3) ration of the partial pressure of arterial oxygen (PaO_2_) to fraction of inspired oxygen (FiO_2_) ≤300 mmHg (1 mmHg = 0.133 kPa). Critically ill cases were defined as having at least one of the following criteria: (1) respiratory failure necessitating mechanical ventilation; (2) shock; (3) failure of other organ systems necessitating care in an intensive care unit [3].

The 58 foreign-imported cases were from 9 countries: 19 (32.8%) from the UK, 9 (15.5%) from Italy, 9 (15.5%) from the USA (15.5%), and the remaining from Spain (6, 10.3%), France (6, 10.3%), Iran (4, 6.9%), Switzerland (3, 5.2%), Austria (1, 1.7%), and Burkina Faso (1, 1.7%). The median age was 29 years (IQR, 20–44). Additionally, 53.4% (31/58) of patients were male and 81.3% (47/58) did not have a clear history of exposure to a confirmed COVID-19 case. The median time from initial symptoms to confirmation was 3 days (IQR, 1–6). Five patients (8.6%) were asymptomatic. The most common symptoms were fever (50%, 29/58) and cough (41.4%, 24/58). High fever presented in only 3 (5.2%) cases. Some (13, 22.4%) patients did not present with either fever or cough. Atypical symptoms, such as nasopharyngeal irritation (19%), headache (10.3%), diarrhea (13.8%), or fatigue (10.3%), were relatively common. Comorbidities were uncommon (7/58, 12.1%) (Table 1).

**Table 1.** Demographic characteristics of imported COVID-19 cases.

Study population	No. (%)
No. of patients	58
Sex, male	31 (53.4)
Age, median (IQR), y	29 (20-44)
<65	56 (96.6)
≥65	2 (3.4)
Ethnic	
Asian	50 (86.2)
White	7 (12.1)
Latino	1 (1.7)
Highest patient temperature, median (IQR), °C	37.4 (36.8-37.9)
<37.3	28 (48.3)
37.3–39	27 (46.6)
≥39	3 (5.2)
Case exposure	
Confirmed COVID-19	11 (19.0)
Suspicious	22 (37.9)
Unkown	25 (43.1)
Time from onset to confirmation, median (IQR), d	3 (1-6)
≤3	35 (60.3)
>3	23 (39.7)
Initial common symptoms	
Fever	29 (50.0)
Cough	24 (41.4)
Nasopharyngeal irritation	11 (19.0)
Dyspnea	2 (3.4)
Fatigue or myalgia	6 (10.3)
Headache	6 (10.3)
Diarrhea	4 (6.9)
Chest imaging, infiltrate	
Normal	24 (41.4)
Pure GGO* (*n *= 34)	24 (70.6)
GGO with reticular and/or interlobular septal thickening (*n *= 34)	12 (35.3)
GGO with consolidation (*n *= 34)	6 (17.6)
Consolidation (*n *= 34)	6 (17.6)
Air bronchogram (*n *= 34)	9 (26.5)
Comorbidities	
Hypertension	7 (12.1)
Diabetes	4 (6.9)
Mood disorder	2 (3.4)
Cardiovascular disease	3 (3.4)
Chronic lung disease	4 (3.4)
Other	3 (5.2)
Initial treatment	
Oxygen therapy	7 (12.1)
Nasal cannula	8 (12.1)
NMV	0 (0)
IMV**	0 (0)
Glucocorticoid	0 (0)
Therapy	
Antibiotic	5 (8.6)
Antiviral	0 (0)
Hydroxychloroquine	57 (98.3)
Thymalfasin	21 (36.2)
Vitamin C	40 (69.0)

*GGO, ground-glass opacity; **IMV, invasive mechanical ventilation.

Only 7 (12.1%) patients required nasal cannula oxygen therapy at admission. Only 5 (8.6%) were administered antimicrobial therapy. Hydroxychloroquine, vitamin C, and thymalfasin were administered as a general treatment.

As of April 4, two severe cases were observed. The first patient was a 54-year-old male identified at admission and who presented with symptoms 15 days prior to testing. Laboratory analysis revealed a low CD4+ T cell count; an *Aspergillus* species was isolated from sputum culture. The second patient was an elderly male (75 years) with no underlying health issues. Hematologic, biochemical, and infection-related indices were normal at admission. He developed a high fever, decreased appetite, diarrhea, and dyspnea within one week after admission. Two severe cases received noninvasive mechanical ventilation (NMV) support and glucocorticoid therapy. By April 4, both patients had been weaned from NMV and were stable. No critically ill cases were observed.

Twenty-two (39.2%, *n* = 56) patients were positive for COVID-19 antibody. Over half (36, 62.1%) of these patients presented a high erythrocyte sedimentation rate and one-third had low pre-albumin (19, 32.8%) level. Approximately one-fourth presented with a mild increase in prothrombin time (17, 29.3%), fibrinogen (16, 27.6%), and D-dimer (12, 20.7%) and lower CD 4+ T cell counts (13, 22.4%).

Based on administrative screening of COVID-19 for all people entering China, many cases were detected in the early-stage and nearly all cases had a low risk of death [2,4–6]. Over 90% of patients presented mild symptoms in an early stage. Most patients (81.0%) did not have a clear contact history. Thus, self-awareness of possible COVID-19 infection was difficult for this population.

Symptoms associated with disease progression [4–6] including high fever, productive cough, and dyspnea were rare (<5% cases). Studies have reported that risk factors related to the development of acute respiratory distress syndrome or death include older age, comorbidities, neutrophilia, low lymphocyte counts, high cytokine levels, coagulation abnormalities or organ dysfunction, and low CD4 T-cell counts [7]. In this study, most patients were young, had a low rate of comorbidities, and showed favourable laboratory indices. Severe cases were rare (3.4%), and the risk of death was low to medium.

The CT findings were consistent with those previously described [8]. Although some studies support routine CT scanning to screen for COVID-19***,*** chest CT imaging was not highly sensitive in this population, mainly because the patients were mostly in early stages. Over 40% of our patients did not present with pulmonary changes in CT scanning; chest CT scans were negative in asymptomatic patients.

Most early-stage cases were from the UK, EU, and USA, indicating an increase in asymptomatic or mild cases in these countries. Our data support that the incoming international epidemic of SARS-CoV-2 originated from covert coronavirus infections [9]. However, hospital-isolation of each mild case may result in depletion of healthcare resources and threaten healthcare resources for patients with other diseases [10]. Additionally, the clinical course and hospitalisation time of patients with COVID-19 were much longer than those of influenza or community-acquired pneumonia. Zhou et al. [11] reported a median duration of viral shedding of 20 days (IQR 17–24), indicating that hospitalised isolation of all patients during an outbreak is not possible. Temporary medical care points such as “Fangcang hospital” or “community isolation point” should be used for these cases [12].

Therefore, immigrants should be continuously monitored for COVID-19 but high-level healthcare measures are not necessary in most cases, as severe disease is unlikely.
